# Effect of TiO_2_ Concentration on Microstructure and Properties of Composite Cu–Sn–TiO_2_ Coatings Obtained by Electrodeposition

**DOI:** 10.3390/ma14206179

**Published:** 2021-10-18

**Authors:** Aliaksandr A. Kasach, Dzmitry S. Kharytonau, Andrei V. Paspelau, Jacek Ryl, Denis S. Sergievich, Ivan M. Zharskii, Irina I. Kurilo

**Affiliations:** 1 Department of Chemistry, Electrochemical Production Technology and Materials for Electronic Equipment, Chemical Technology and Engineering Faculty, Belarusian State Technological University, Sverdlova 13a, 220006 Minsk, Belarus; inorgchem@belstu.by; 2 Jerzy Haber Institute of Catalysis and Surface Chemistry, Polish Academy of Sciences, Niezapominajek 8, 30-239 Krakow, Poland; 3 Physical and Chemical Investigations Methods Center, Belarusian State Technological University, Sverdlova 13a, 220006 Minsk, Belarus; pospelov.ip@gmail.com; 4 Institute of Nanotechnology and Materials Engineering, Faculty of Applied Physics and Mathematics, Gdansk University of Technology, 80-233 Gdansk, Poland; jacek.ryl@pg.edu.pl; 5 Department of Biotechnology, Organic Substances Technology Faculty, Belarusian State Technological University, Sverdlova 13a, 220006 Minsk, Belarus; sergievich@belstu.by; 6 Department of Physical, Colloid and Analytical Chemistry, Organic Substances Technology Faculty, Belarusian State Technological University, Sverdlova 13a, 220006 Minsk, Belarus; kurilo@belstu.by

**Keywords:** copper–tin alloy, electrodeposition, composite coating, titania, antibacterial properties, corrosion

## Abstract

In this work, Cu–Sn–TiO_2_ composite coatings were electrochemically obtained from a sulfate bath containing 0–10 g/L of TiO_2_ nanoparticles. The effect of TiO_2_ particles on kinetics of cathodic electrodeposition has been studied by linear sweep voltammetry and chronopotentiometry. As compared to the Cu–Sn alloy, the Cu–Sn–TiO_2_ composite coatings show rougher surfaces with TiO_2_ agglomerates embedded in the metal matrix. The highest average amount of included TiO_2_ is 1.7 wt.%, in the case of the bath containing 5 g/L thereof. Composite coatings showed significantly improved antibacterial properties towards *E. coli* ATCC 8739 bacteria as compared to the Cu–Sn coatings of the same composition. Such improvement has been connected with the corrosion resistance of the composites studied by linear polarization and electrochemical impedance spectroscopy. In the bacterial media and 3% NaCl solutions, Cu–Sn–TiO_2_ composite coatings have lower corrosion resistance as compared to Cu–Sn alloys, which is caused by the nonuniformity of the surface.

## 1. Introduction

Bacterial infections and their complications cause a decline in the life quality and death of millions of patients around the world every year. The most common route of transferring pathogenic microorganisms is direct human contact with contaminated contact surfaces (fomites). That is an urgent problem in public places, for example, shopping centers, educational facilities, hospitals, and office buildings, since their microclimate promotes a rapid growth and transmission of microorganisms on metal handrails and doorknobs [1,2,3,4]. Moreover, many types of germs can survive on indoor surfaces for several days, with some of them being able to survive for longer than one month. In this regard, potentially contaminating surfaces must provide antibacterial and anticorrosive protection while maintaining high decorative and mechanical properties.

Recently, copper and copper alloys have attracted increased attention due to their pronounced antibacterial activity against many germs [5,6,7,8,9,10,11]. Van Doremalen et al. reported [12] that copper surfaces are the most effective in the suppression of the SARS-CoV-2 and SARS-CoV-1 coronaviruses activity with a destruction time of 4 h. For comparison, they remained active on plastic and stainless-steel surfaces for up to 72 h. It is generally accepted [13] that the use of copper-based fomites can reduce the number of infections caused by SARS-CoV-2 and other viruses. Hutasoit et al. proposed a cold-spray coating of copper on in-use steel parts to alleviate the tendency of SARS-CoV-2 spreading, with 96% of the virus inactivated within 2 h [14]. Copper and copper-based coatings can also be effectively used against antibiotic-resistance hospital bacteria, like *Escherichia coli* (*E. coli*), *Bacillus subtilis*, and *Staphylococcus epidermidis* (*S. epidermidis*) [11,15]. However, copper has low wear resistance, quickly tarnishes, and loses its decorative appearance. On the contrary, copper-based alloys have high mechanical properties and good corrosion resistance. It is known that tin bronzes obtained by metallurgical methods have satisfactory antibacterial activity [7,9,16]. However, from the economic point of view, it is more favorable to apply a top finishing layer of an active coating on the goods. For this reason, electrodeposition of functional top coatings is a recent trend in the management of potentially contaminated environments. During galvanic deposition of alloys, it is possible to obtain phases, which are stable at room temperature but do not correspond to the phase diagrams (intermetallics, solid solutions, and high-temperature phases) [17]. For this reason, galvanic and metallurgical alloys of the same quantitative composition can differ significantly in their physical-mechanical and physical-chemical properties.

Galvanic Cu–Sn coatings containing 10–20 wt.% of tin are similar in their physical-mechanical and corrosion properties to nickel coatings, however, unlike the latter, they are hypoallergenic and have lower cost [18,19]. Electrochemical deposition of Cu–Sn alloys can be performed from the sulfuric acid electrolytes, which are the most versatile and easy-to-prepare type of plating bath [20,21,22]. Co-deposition of copper and tin from these electrolytes can occur at potentials that are more positive than the equilibrium potential of the Sn^2+^/Sn system (−0.14 V) due to the underpotential deposition (UPD) of tin [20]. Utilization of sulfuric acid electrolyte allows obtaining high-quality Cu–Sn coatings containing 6–18 wt.% of tin [22]. There are several studies reporting antibacterial properties of Cu–Sn coatings [7,9]. However, these reports are mostly focused on the antibacterial effect of Cu–Sn coatings containing 4–6 wt.% of tin.

Further improvement of such coatings can be achieved by the co-deposition of a metal matrix with the second-phase reinforcement particles, forming nanocomposites with enhanced functional properties and extended potential applications [23,24,25]. The use of electrochemical deposition for this purpose has many important advantages, such as low energy consumption, uniform distribution of reinforcing particles in a metal matrix, and better bonding between particles and metal matrix [24,26,27]. Composite coatings Cu–Sn–SiC [28], Cu–Sn–graphite–Al_2_O_3_ [29], and Cu–Sn–TiO_2_ [25,30,31] with improved tribological and physicomechanical properties were obtained electrochemically. Titanium(IV) oxide due to its physical-mechanical and photocatalytic properties, as well as chemical resistance in corrosive media, is widely used as a hardening phase in the deposition of composite coatings [32,33,34,35,36,37,38,39]. Galvanic coatings modified with TiO_2_ nanoparticles can exhibit photocatalytic properties, which is well demonstrated in the literature [33,34,35]. Improved tribological properties of Cu–Sn–TiO_2_ coatings were reported in [25,30,31]. Nevertheless, to our best knowledge, the effect of TiO_2_ particles on the co-deposition of copper and tin, as well as on the corrosion and antibacterial properties of such nanocomposites has not been studied widely in the literature.

In this work, we aimed to investigate the process of electrochemical deposition of Cu–Sn–TiO_2_ coatings from a sulfuric acid electrolyte. The effect of the concentration of TiO_2_ nanoparticles in the electrolyte on the microstructure, qualitative and quantitative composition, physical-mechanical, antibacterial, and corrosion properties of the formed coatings was studied.

## 2. Materials and Methods

### 2.1. Electrodeposition of Composite Coatings

**Figure 1:**
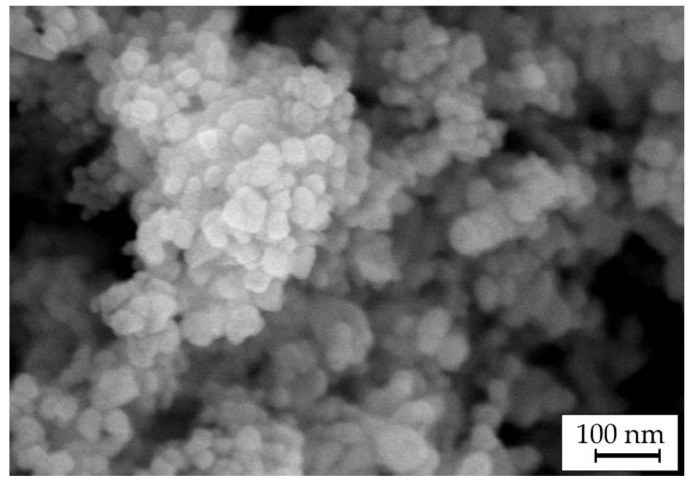
SEM image of Degussa P25 TiO_2_ powder used in this work.

The electrochemical deposition of Cu–Sn–TiO_2_ composite coatings was performed from a sulfuric acid bath of the composition listed in Table 1. All solutions were prepared with 18.2 MΩ cm deionized water (Polwater, Krakow, Poland) and reagents of chemically pure grade (Belreachim, Minsk, Belarus). The pH of the as-prepared electrolyte was <1. As a second phase, TiO_2_ nanoparticles (Degussa aeroxide P25) were introduced into the electrolyte in amounts of 0, 1, 5, and 10 g/L. Hereafter, the coatings obtained from these electrolytes are labeled as Ti-0, Ti-1, Ti-5, and Ti-10, respectively. Figure 1 shows the scanning electron microscopy (SEM) image of the used TiO_2_ powder with the particle sizes ranging from 30 to 50 nm.

Before electrodeposition, as-prepared solutions were ultrasonically treated (power of 50 W) using an UP 200 Ht ultrasonic homogenizer (Hielscher Ultrasonics GmbH, Teltow, Germany) for 20 min to provide deagglomeration and better dispersion of TiO_2_ particles in the volume of the electrolyte. In the course of the deposition, the electrolyte was mechanically stirred every 5 min for 10 s (stirring rate 400 rpm). Cathodic deposition of coatings was performed in the potentiostatic mode at 20 °C. The deposition time varied depending on the applied potential to obtain coatings with a thickness of 10 μm. The values of the cathodic deposition potentials are indicated in the text of the contribution in the scale of a standard hydrogen electrode (SHE). A copper-foiled dielectric with a surface area of 8 cm^2^ was used as the cathode and an M0 copper plate was used as the anode. The distance between the cathode and the anode was 40 mm. Before experiments, cathodes were chemically treated according to the State Standard GOST 9.305-84. The treatment included degreasing in the solution containing 15 g/L Na_2_CO_3_, 30 g/L Na_3_PO_4_·12H_2_O, and 5 g/L Sintanol DS10 with further activation in 0.1 M H_2_SO_4_.

Cathodic polarization curves and chronopotentiograms in the studied electrolyte were recorded in a standard three-electrode glass electrochemical cell using an AUTOLAB PGSTAT302N potentiostat/galvanostat (Metrohm Autolab B.V., Utrecht, The Netherlands). The linear potential sweep rate during polarization measurements was 1 mV/s. The working electrode was a copper-foiled dielectric with an area of 1 cm^2^; a silver/silver chloride was used as the reference electrode, and an M0 copper plate served as the counter electrode.

### 2.2. Morphology and Structural Analysis

The morphology and elemental composition of the formed coatings were determined using a JSM-5610 LV (JEOL Ltd., Tokyo, Japan) scanning electron microscope equipped with an EDX JED-2201 JEOL elemental analysis system. The recalculation of the mass content of titanium to the titanium dioxide was carried out according to the formula:

(1) $$\omega ({\mathrm{TiO}}_{2})=\omega (\mathrm{Ti})M({\mathrm{TiO}}_{2})/A(\mathrm{Ti})$$

where ω(TiO_2_) is the mass content of titanium dioxide in the coating (wt.%); ω(Ti) is the weight content of titanium in the coating according to EDX data (wt.%); *M*(TiO_2_) is the molar mass of TiO_2_ (g/mol), and *A*(Ti) is the atomic mass of titanium (a. e. m.).

The structural analysis of coatings was performed by X-ray diffraction (XRD) using a PANalytical Cubix X’Pert Pro X-ray (Malvern Panalytical B.V., Almelo, The Netherlands) diffractometer using CuKα radiation (λ = 1.5418 Å). Diffraction patterns were recorded at a scan rate of 2 deg/min with a 0.1 deg. step. The obtained diffraction patterns were processed using the Match! software and the COD (Crystallography Open Database) reference base.

The X-ray photoelectron spectroscopy (XPS) measurements were done using an ESCALAB 220 XL XPS spectrometer (Thermo Fisher Scientific, Waltham, MA, USA) featuring a monochromatic Al Kα X-ray source (1486.6 eV). A pass energy of 20 eV was applied. The charge compensation was assured through low-energy electron and low-energy Ar+ ions bombardment of the sample, with the final peak calibration at adventitious carbon C1s (284.6 eV).

### 2.3. Antibacterial Performance

Antibacterial properties of the obtained coatings were determined using the classical Koch method by counting living bacteria colonies after their contact with the examined surfaces. The test cultures were the sanitary indicative *Escherichia coli* (*E. coli*) ATCC 8739. Before inoculation of the bacteria, the test samples were sterilized in ethanol (70%) for 1 h and then dried under UV irradiation. Overnight culture of *E. coli* was diluted to a concentration of ~10^6^–10^7^ CFU/mL and inoculated (50 µL) onto the surface of the studied samples. The samples were covered with a sterile film to ensure the spreading of the bacterial culture over the surface and incubated for 45 or 90 min. After incubation, the samples (with a film) were washed with 10 mL of saline solution containing Triton x-100 (0.001 vol.%). Undiluted wash liquid was plated on a solid medium (nutrient agar). The inoculations were incubated in such conditions for 24 h at a temperature of 30 °C, after which the colony-forming units were counted. All corrosion and antibacterial experiments reported in this paper were at least triplicated on independent samples.

### 2.4. Electrochemical Measurements

In electrochemical experiments, a platinum mesh was used as the counter electrode and a saturated silver/silver chloride electrode served as a reference. As a model corrosive medium, 3% NaCl solution and bacterial media were used. The composition of the bacterial media is provided in Table S3 in the Supplementary Information. The polarization curves of the obtained coatings were recorded in the potential range from −200 mV to +200 mV relative to the open-circuit potential (OCP). The electrochemical impedance spectroscopy (EIS) spectra were recorded at the OCP value in the frequency range from 10^5^ to 10^–2^ Hz, the ac potential amplitude of 10 mV, and 7 measured points per frequency decade. The consistency of the EIS data was evaluated using the Kramers–Kronig transformation procedure available in the potentiostat software. The analysis of the EIS spectra, selection of equivalent circuits, and fitting of their parameters were performed in the ZView 3.2c software.

## 3. Results and Discussion

### 3.1. Electrodeposition of Cu–Sn–TiO_2_ Composite Coatings

**Figure 2:**
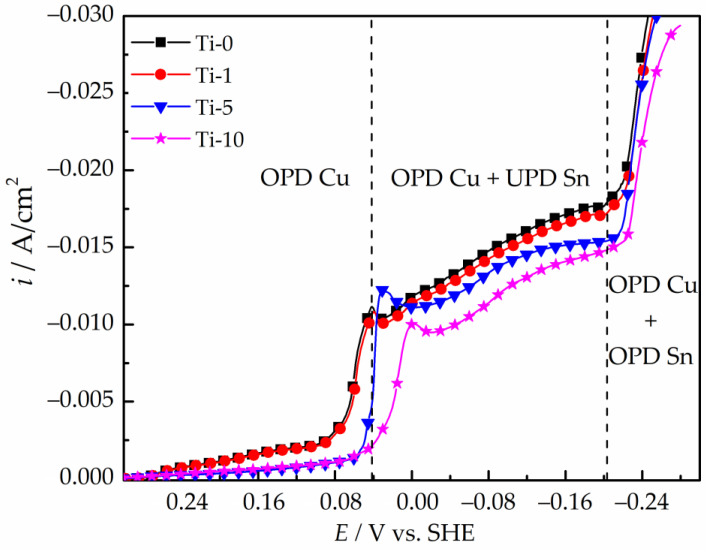
Cathodic polarization curves of copper electrode in sulfate electrolytes of Cu–Sn–TiO_2_ deposition.

Figure 2 presents polarization curves of the copper electrode obtained in the studied electrolytes containing 0–10 g/L TiO_2_. The introduction of TiO_2_ nanoparticles into the electrolyte leads to a shift of the polarization curves to the electronegative region. All the obtained polarization dependences can be divided into three areas. In the range of potentials from 0.30 to 0.00 V, only copper(II) ions are discharged at the cathode. When potentials more negative than 0.04–0.00 V are reached, the polarization curves show a monotonic increase in the current density, which is caused by the UPD of Sn. At potentials lower than −0.20 V, the slope of the current-voltage dependence changes, which is caused by the onset of the discharge of stannous ions under the overpotential deposition (OPD). The obtained polarization curves revealed that the introduction of TiO_2_ particles into the studied bath leads to only slight inhibition of the discharge of copper(II) ions and a decrease in the overall rate (current density) of processes occurring on the electrode in the potential range from 0.00 to −0.20 V.

The electrochemical deposition of copper and tin in sulfuric acid electrolytes occurs under the diffusion control of the discharge of copper(II) ions [20,21,30,35,40]. For this reason, a decrease in the cathodic current densities in the electrolytes containing TiO_2_ nanoparticles seen in Figure 2 can be due to the following reasons:

(i) according to the Guglielmi model of the electrochemical deposition of composite coatings [40], TiO_2_ nanoparticles can physically adsorb on the electrode surface during electrolysis, thereby reducing the active electrode area involved in the electrochemical process. This leads to a decrease in the current response at an applied potential;
(ii) since diffusion is the limiting stage of the discharge of copper(II) ions in acidic electrolytes, TiO_2_ particles dispersed in the electrolyte could prevent diffusion and migration of copper and stannous ions from the bulk solution to the electrode surface.

**Figure 3:**
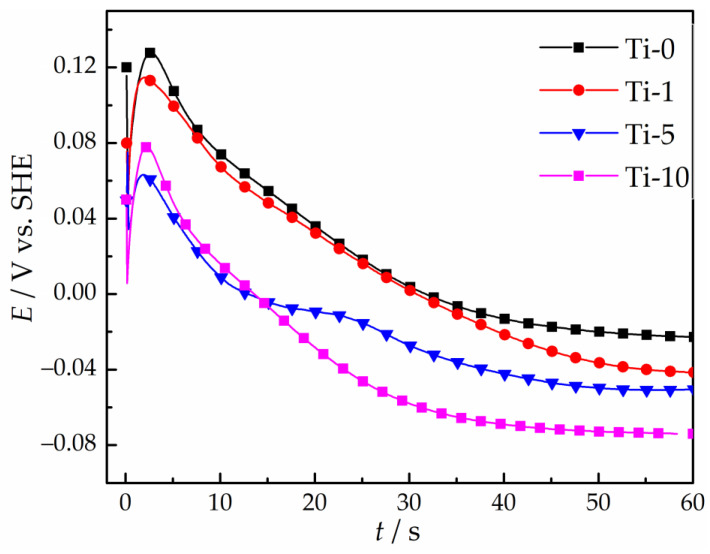
Chronopotentiograms of copper electrode in sulfate solutions obtained at the cathodic current density of −0.015 A/cm^2^.

To study the effect of TiO_2_ nanoparticles on the galvanostatic deposition of Cu–Sn–TiO_2_ coatings, chronopotentiograms of the copper electrode in the examined solutions were obtained at a cathodic current density of −0.015 A/cm^2^ (Figure 3).

At the initial moment of deposition (from 1 to 2 s), the chronopotentiograms exhibit potential maxima corresponding to the overvoltage of the formation of the first copper clusters [20]. After reaching the maximum, the electrode potential gradually shifts to the electronegative region, which is caused by a progressive reduction of copper ions in the cathode interface. The subsequent plateau of the electrode potential indicates that copper ions discharge at the limiting diffusion current and the UPD of tin is initiated [20,41]. As the content of TiO_2_ nanoparticles in the electrolyte increases, the dependences shift to more negative potentials, which may indicate a decrease in the fraction of the current consumed to the discharge of copper(II) ions and subsequent increase in the partial current of the tin deposition. Therefore, this effect of TiO_2_ particles must be considered during electrodeposition under galvanostatic conditions, since increasing the electrode potential will lead to an increase in the tin content in the alloy. This may complicate a comparative assessment of the corrosion and antibacterial properties of Cu–Sn and Cu–Sn–TiO_2_ coatings.

To assess the effect of the TiO_2_ nanoparticles in the electrolyte on the microstructure and grain size of the formed coatings, electrolysis was carried out in the potentiostatic mode at a deposition potential of −0.05 V.

### 3.2. Microstructure and Elemental Composition

**Figure 4:**
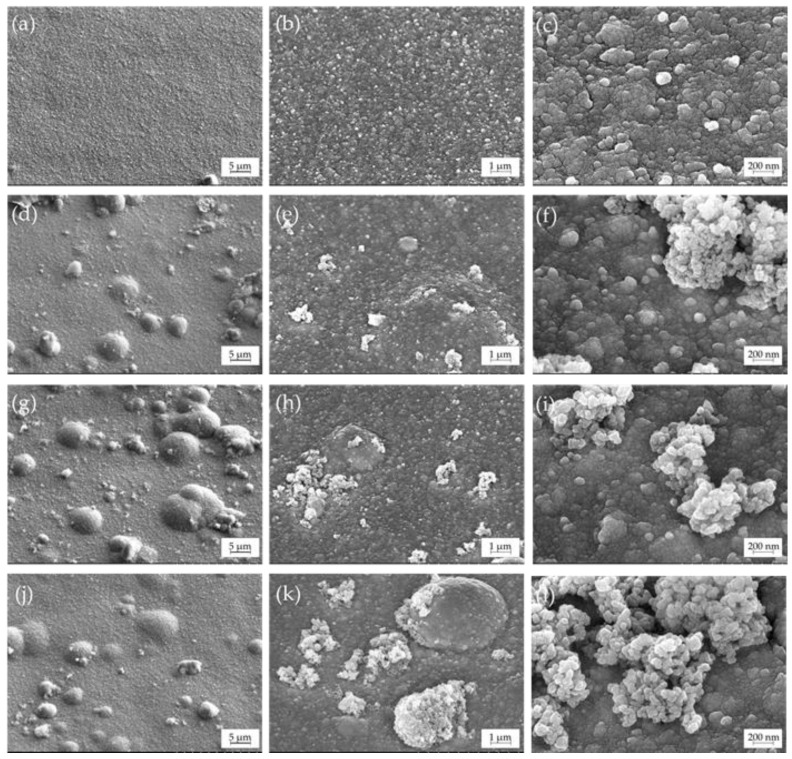
SEM images of Cu–Sn (**a**–**c**) and Cu–Sn–TiO_2_ coatings Ti-1 (**d**–**f**), Ti-5 (**g**–**i**), and Ti-10 (**j**–**l**) deposited at the cathodic potential of −0.05 V.

Figure 4 shows SEM images of the obtained coatings. Homogeneous fine-grained coatings were formed from the electrolyte without TiO_2_ particles (Figure 4a). Obtained coatings have good adhesion to the substrate without visible delamination at the coating/substrate interface (cross-sectional images, Figure S1 in the Supplementary Information). The grain size of the formed alloy varies from 20 to 70 nm. The introduction of TiO_2_ particles in the electrolyte did not significantly change the grain size of the metal matrix, however, it did noticeably change the microstructure (Figure 4b–d). It is clearly seen that many sphere-shaped features with sizes between 1 and 10 μm were formed on the surface of Ti-1, Ti-5, and Ti-10 coatings (Figure S1 in the Supplementary Information). Nonuniform inclusions of 200–700 nm in size, which were not observed in the case of Cu–Sn coating, are also noticeable in the microstructure. An increase in the content of TiO_2_ particles in the electrolyte resulted in higher inhomogeneity of the surface and an increased number of agglomerates in the alloy matrix. The formation of globular deposits could be ascribed either to theTiO_2_ particles embed into the metal matrix and then covered by a next layer of the coating or to the negative effect of the second-phase particles on the current distribution over the electrode surface. Introduction of TiO_2_ also decreased luster of the coatings but significantly improved their microhardness (Table S1 in the Supplementary Materials).

**Figure 5:**
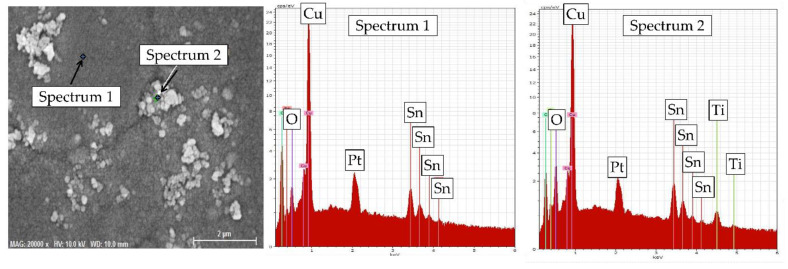
Point EDX analysis of Ti-1 coating surface. The signal from Pt on EDX spectra originates from the top sputtered layer.

The point EDX analysis of the two characteristic morphology regions on the surface of Ti-1 coating is shown in Figure 5. The results confirmed that fine smooth areas of the surface are Cu–Sn alloy with the stacked granules being TiO_2_ particles.

Table 2 reports the EDX chemical composition of the coatings electrodeposited from the electrolytes with varying TiO_2_ content. High-quality yellow coatings were formed at a deposition potential of −0.05 V (region of Sn UPD). As expected from the selected electrodeposition mode (Figure 3), coatings have almost equal content of tin (11.3–11.5 wt.%) with the Ti-1 coating containing 10.8 wt.% of tin. The maximum average amount of TiO_2_ was observed in the Ti-5 coating (1.7 wt.%). It can be explained by higher agglomeration and, consequently, lower sedimentation stability of the electrolyte containing 10 g/L TiO_2_. However, we cannot exclude some variation of the TiO_2_ content in the bulk of the examined coatings.

**Figure 6:**
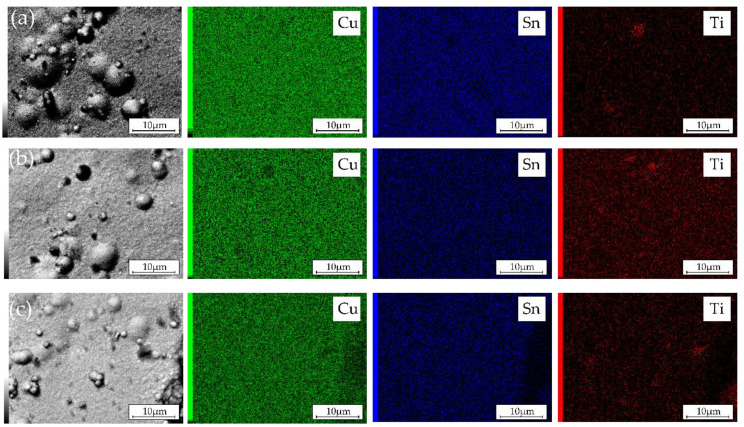
SEM images (to the left) and corresponding EDX elemental maps of Ti-1 (**a**), Ti-5 (**b**), and Ti-10 (**c**) Cu–Sn–TiO_2_ composites obtained at −0.05 V.

The EDX element distribution maps over the surface of the electrodeposited composite coatings are shown in Figure 6. They illustrate that Cu and Sn are uniformly distributed over the surface regardless of the concentration of TiO_2_ particles in the electrolyte. During the electrodeposition process, TiO_2_ particles are embedded into the metal matrix as agglomerates with sizes varying from sub- to several microns.

### 3.3. XRD and XPS Analysis

**Figure 7:**
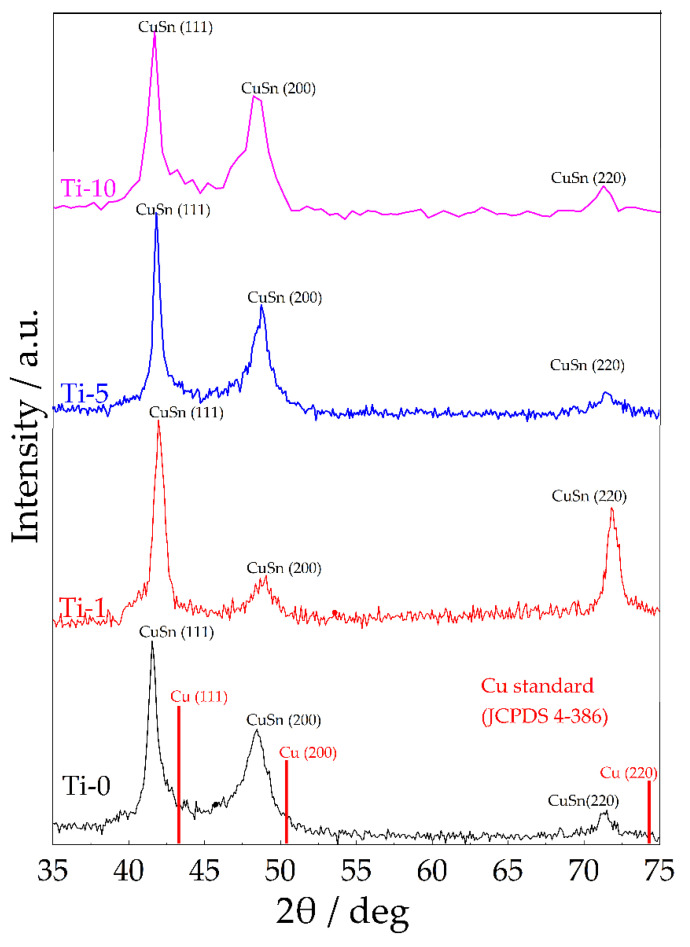
XRD patterns of Cu–Sn and Cu–Sn–TiO_2_ coatings (thickness 10 µm).

Figure 7 shows XRD patterns of the obtained coatings. As can be seen from the data obtained, the main reflections of the obtained coatings are shifted to smaller angles as compared to the pure Cu phase (JCPDS 4-368). This indicates an increase in the intercrystallite distance of the metal matrix due to the introduction of tin atoms into the copper crystal lattice. This shift was lower for the Ti-1 coating, as it has the lowest amount of tin (Table 2). All coatings have the fcc crystal structure with the preferred orientation in (111) plane. The formation of a single-phase solid solution is consistent with our previous studies [20].

**Figure 8:**
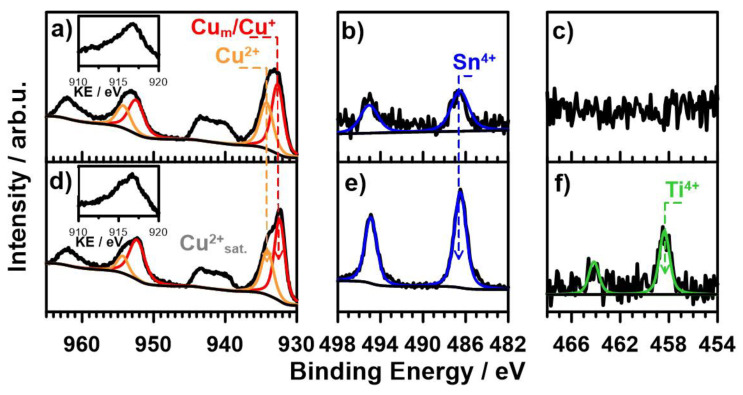
XPS spectra of (**a**–**c**) Cu–Sn and (**d**–**f**) Cu–Sn–TiO_2_ coatings. High-resolution spectra recorded in the binding energy range of (**a**,**d**) Cu2p, (**b**,**e**) Sn3d, and (**c**,**f**) Ti2p. The inset in (**a**) shows Cu_LMM_ Auger spectra.

The XPS analysis was performed to analyze the modification of Cu–Sn surface chemistry through TiO_2_ embedding into the coating. The results of high-resolution XPS analysis recorded in the core-level binding energy range of Cu2p, Sn3d, and Ti2p spectra are summarized in Figure 8.

The recorded Cu2p spectra reveal complex copper chemistry, two peak doublets were used for the deconvolution process with Cu2p_3/2_ peaking at 932.7 and 934.1 eV, respectively. The primary of the two components, located at lower binding energies lies in the energy range characteristic of metallic copper and copper (i) oxides. To distinguish them further, the supplementary Cu_LMM_ Auger electron spectra were recorded with the kinetic energy peaking at 916.8 eV for both studied samples. These results suggest that Cu_2_O is the primary copper constituent within the discussed XPS spectra, confirming the proposed corrosion mechanism (see Equation (6)). The second Cu2p component is located at the binding energies typical for Cu^2+^ and is most likely present in the form of Cu(OH)_2_, a conclusion drawn based on the peak binding energy value and characteristic shape of the Cu^2+^ satellite features [42]. Interestingly, the addition of TiO_2_ does not significantly alter the Cu^+^:Cu^2+^ ratio.

The XPS data confirmed the presence of Ti^4+^ species at the surface of the analyzed Cu–Sn–TiO_2_ sample, through the appearance of Ti2p3/2 peak at approx. 458.4 eV. Furthermore, tin oxidation in line with Equation (3) was confirmed through identification of Sn^4+^ Sn3d_5/2_ peak at 486.6 eV. The addition of TiO_2_ to the studied Cu–Sn coating did not alter tin surface chemistry, but, interestingly, a consequence was a higher surface Sn:Cu ratio (12.4% in the case of Cu–Sn–TiO_2_ compared to 2.6% for Cu–Sn coating). This can suggest that the surface of composite coatings is enriched in the corrosion products of tin, which is anodic component in the Cu–Sn system. The detailed surface composition examined by XPS is summarized in Table S2 in the Supplementary Information.

### 3.4. Antibacterial Performance

**Figure 9:**
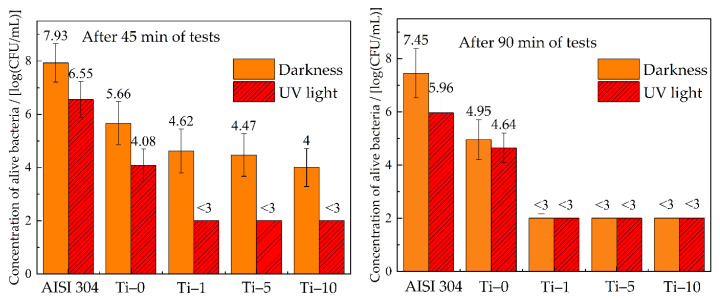
Antibacterial properties of obtained coatings towards *E. coli* ATCC 8739.

The antibacterial activity of the studied coatings was determined relative to the sanitary indicative bacteria *E. coli* ATCC 8739. Stainless-steel coupons (AISI 304) were used as a reference. The results of the bacterial tests are summarized in Figure 9. The concentration of viable cells in the initial suspension was 9.8 × 10^6^ CFU/mL. In the case of the reference sample, an increase in the number of bacteria colonies to 8.6 × 10^7^ CFU/mL was observed after 45 min of the sample contact with the bacteria without UV treatment. This indicates that the stainless-steel surface provides favorable conditions for the reproduction of microorganisms. In turn, all obtained copper-based coatings showed pronounced antibacterial effect. The concentration of bacteria decreased by 1–2 orders of magnitude after 45 min of the contact with the Ti-0 sample. Coatings modified with TiO_2_ particles showed improved antibacterial properties in comparison with Cu–Sn coatings. After 45 min of contact with the surface of Cu–Sn–TiO_2_ coatings, the number of active *E. coli* colonies decreased by 3 orders of magnitude, and after 90 min of testing, it was below the detection limit.

Evaluation of antibacterial properties under UV irradiation showed that this type of treatment decreased the number of active colonies on the surface of Cu–Sn coatings almost two times as compared to the data without UV treatment after 90 min of contact. In the case of Cu–Sn–TiO_2_ composite coatings, the number of bacteria colonies was below the detection limit after 45 min of UV treatment. High antibacterial activity of Cu–Sn–TiO_2_ coatings under UV treatment is explained by the presence of TiO_2_ particles on their surface. These particles, being photocatalytically active, generate active forms of oxygen, which have a strong oxidation stress on the bacteria cells [43].

The antibacterial properties of copper and its alloys are usually connected to the release of copper ions, which have a strong oxidative effect on the membrane and/or wall of the bacterial cell [7,8,9,10]. It leads to the damage of the cell membrane and further to disruption of the expression of genetic material. In this regard, the antibacterial activity of the coatings is mainly connected with the concentration of copper ions at the coating/electrolyte interface. In turn, concentration of copper ions will strongly depend on the corrosion rate of the coating in the operating environments. For this reason, a detailed evaluation of the corrosion resistance of the formed composites was performed in 3% NaCl solutions and bacterial media.

### 3.5. Corrosion Properties

**Figure 10:**
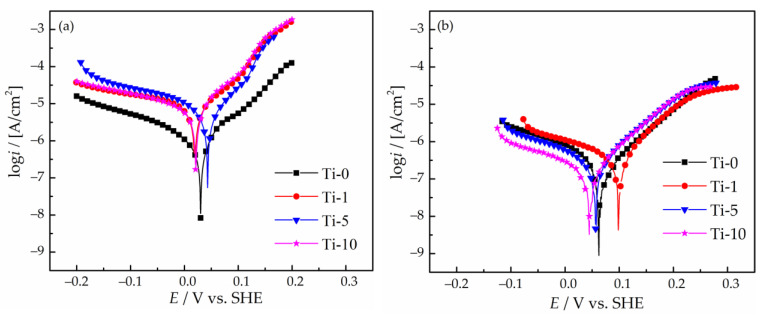
Potentiodynamic polarization curves of Cu–Sn and Cu–Sn–TiO_2_ coatings after 15 min exposure to 3% NaCl solution (**a**) and bacterial media (**b**).

The corrosion performance of the obtained Cu–Sn and Cu–Sn–TiO_2_ coatings in the 3% NaCl solution was evaluated by polarization and EIS studies. The polarization curves obtained after 15 min exposure of the coatings to the 3% NaCl solution are shown in Figure 10a. The electrochemical parameters of the corrosion process calculated from the potentiodynamic polarization data are summarized in Table 3.

Analysis of the polarization curves showed that the corrosion potential, *E*_corr_, for Ti-0 sample is 0.030 V. For the Ti-1 and Ti-10 coatings *E*_corr_ it slightly shifted to the cathodic region reaching the value of ca. 0.020 V. Oppositely, for the Ti-5 coating, a shift to a more noble potential of 0.043 V was observed. Such a small variation in *E*_corr_ can be caused by the surface passivation and almost equal Cu-to-Sn ratio in the coatings. The calculated value of the corrosion current density, *i*_corr_, for the Ti-0 sample is 1.2 × 10^–7^ A/cm^2^. The introduction of TiO_2_ nanoparticles into the deposition bath deteriorated the corrosion stability of all TiO_2_-containing composites, which are characterized by 7–9 times higher *i*_corr_ values as compared to the Ti-0 coating.

Furthermore, the solution used for bacterial testing has a different composition (Table S3 in the Supplementary Information). The results of the corrosion experiments in this medium showed that the corrosion rate was generally lower than in 3% NaCl solution (Figure 10b and Table 3). Thus, it can be expected that the antibacterial performance of the obtained composites in the exploitation conditions could be further improved due to a faster release of copper ions from the coating.

**Figure 11:**
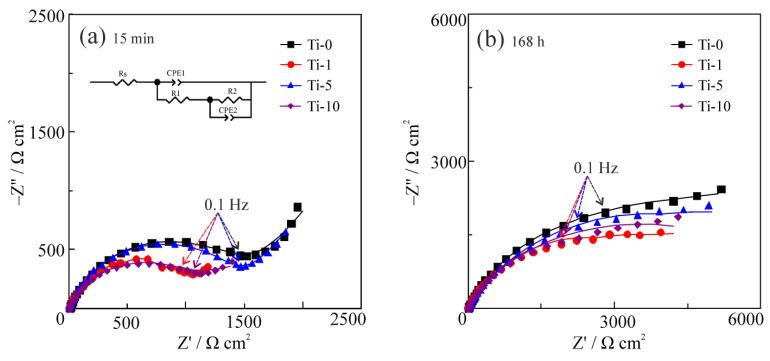
Nyquist EIS plots of Cu–Sn and Cu–Sn–TiO_2_ coatings after 15 min (**a**) and 168 h (**b**) of exposure to 3% NaCl solution. Symbols correspond to experimental data and lines are results of curve fitting. Equivalent circuit used for data evaluation is shown as an inset in (**a**).

To further evaluate the dynamics of the corrosion performance of the examined coatings, EIS spectra were obtained after 15 min (Figure 11a) and 168 h (Figure 11b) of their corrosion in 3% NaCl solution. In both cases, two time constants in the form of suppressed semicircles can be distinguished in the Nyquist plots of all samples. The semicircle at high and medium frequencies characterizes the coating surface and the semicircle at low frequencies characterizes the coating/substrate interface. The presence of two time constants implicitly confirmed the heterogeneity of the formed coatings. Analysis of the EIS spectra showed that the Ti-0 coating has higher values of the impedance modulus than the coatings modified with TiO_2_ particles. A significant increase in the impedance modulus of all the samples was observed after 168 h of exposure to the corrosive environment (Figure 11b), which is due to the formation and compaction of a passive film of the corrosion products on the surface.

To quantitatively describe the obtained impedance spectra, the equivalent circuit shown in Figure 11a was used. Here, *R*_1_ corresponds to the electrolyte resistance; *R*_2_ is the resistance of the surface layer of corrosion products; CPE_1_ describes the capacitive response of the layer of corrosion products; *R*_3_ is the charge transfer resistance; CPE_2_ is the capacitive response of the electric double layer. The constant phase element (CPE) was used due to the significant heterogeneity of the coating surface. The impedance of the CPE element is given by:

(2) $$Z_{\mathrm{CPE}}=\frac{1}{Y{\left(jw\right)}^{n}}$$

where *Y* is the CPE constant and *n* is the mathematical factor.

The results of the data fitting are presented in Table 4. Comparison of the corrosion resistance of the coatings was performed based on the values of the polarization resistance, *R*_p_, which for the used equivalent circuit was calculated as *R*_p_ = *R*_2_ + *R*_3_. It was found that regardless of the exposure duration, the composite coatings have lower *R*_p_ values than the Ti-0 coating. This result is in good agreement with the polarization studies (Figure 10).

**Figure 12:**
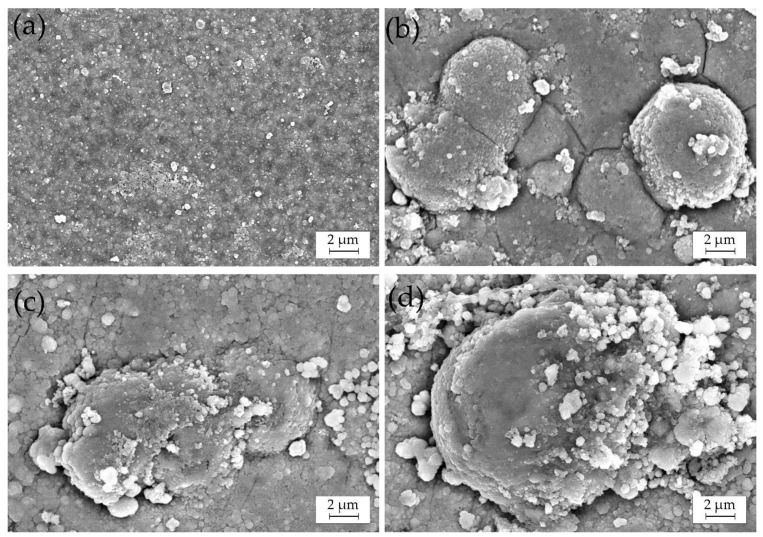
SEM images of Ti-0 (**a**), Ti-1(**b**), Ti-5 (**c**), and Ti-10 (**d**) after 168 h of corrosion experiment in 3% NaCl solution.

SEM micrographs of the Cu–Sn and Cu–Sn–TiO_2_ coatings after 168 h of immersion corrosion tests in the 3% NaCl solution are shown in Figure 12. Numerous adsorbed particles, most probably insoluble corrosion products, were seen on the surface of the Ti-0 coating (Figure 12a). Despite this noticeable uniform change, no pronounced local corrosion attack was observed. In the case of Cu–Sn–TiO_2_ composites (Figure 12b–d), the local corrosion attack was mostly concentrated around globular formations in the microstructure of the coatings. Our SEM/EDX analysis (Figure 5 and Figure 6) confirmed that those are surface areas where TiO_2_ particles are embedded into the alloy matrix. Such microstructure is characterized by significant heterogeneity and microdefects, which, in turn, can act as initiation sites of the local corrosion attack. The EDX analysis confirmed the presence of insoluble chloride-containing corrosion products on their surface (Figure S1 in the Supplementary Information). Summarizing, the results of corrosion tests in 3% NaCl solution indicated that TiO_2_ particles can slightly decrease the corrosion resistance of the Cu–Sn matrix.

The corrosion process of copper–tin alloys in chloride-containing media can be described by the following mechanism [38,44,45,46]. Tin is a more electronegative component of the Cu–Sn alloy. Therefore, oxidation of tin at the Cu–Sn alloy/electrolyte interface will occur first with the formation of a SnO_2_ layer [44,45]:

Sn + 2H_2_O − 4*e* → SnO_2_ + 4H^+^. (3)

After this, the dissolution of the main component of the alloy (copper) in a chloride-containing medium can proceed through the stage of the Cu^+^ ion formation according to the reactions [44,45]:

(4) $$\mathrm{Cu}\;-e\to {\mathrm{Cu}}^{+}\;$$

(5) $${\mathrm{Cu}}^{+}{+\mathrm{Cl}}^{-}\to \;\mathrm{CuCl}.$$

These corrosion products form a low-soluble dense adsorbed surface film [46]. At high concentrations of chloride ions in the near-electrode layer, the formation of CuCl^–^ ions is also possible. They can further interact with water molecules, forming a layer of poorly soluble Cu_2_O on the sample surface [44,45]:

(6) $${\mathrm{CuCl}}^{-}+H_{2}O\to {\;\mathrm{Cu}}_{2}O+2H^{+}+4{\mathrm{Cl}}^{-}.$$

In general, the incorporation of particles into electrodeposited metal coatings is reported to improve their corrosion resistance [23,47,48]. However, contradicting results on the corrosion resistance of the electrochemically deposited Cu–Sn–TiO_2_ composite coatings are reported in the literature. Ying et al. reported significantly improved corrosion resistance of Cu–Sn–TiO_2_ coatings due to the improved coating structure [30]. In turn, Gao et al. observed slightly improved corrosion resistance of Cu–Sn–Zn–1 g/L TiO_2_ coatings, while those deposited from the bath containing 5 g/L TiO_2_ were characterized by higher *i*_corr_ [32]. Our results showed that all TiO_2_-containing composites are characterized by lower corrosion resistance as compared to the Cu–Sn alloy, probably due to the agglomeration of TiO_2_ particles. This results in the nonuniformity of the coating surface. The highest corrosion resistance among the composite coatings was shown by the Ti-5 sample, for which the *R*_p_ values are comparable with those for the Ti-0 coating. This composite is also characterized by the best uniformity of the second-phase particles distribution over the surface. Another important factor is that TiO_2_ agglomerates embedded in the Cu–Sn matrix can form local microcathodes on the surface of the alloy, causing selective dissolution of the metal matrix around them. However, the main corrosion parameters of the obtained composites were still acceptable for their industrial application. Moreover, such corrosion behavior is favorable in terms of their antibacterial performance.

Furthermore, the solution used for bacterial testing has a different composition (Table S3 in the Supplementary Information). The results of the corrosion experiments in this medium showed that the corrosion rate was generally lower than in 3% NaCl solution (Figure 10 and Table 3). Thus, it can be expected that the antibacterial performance of the obtained composites in the exploitation conditions could be further improved due to a faster release of copper ions from the coating. Summarizing, the use of topcoat Cu–Sn–TiO_2_ coatings in public areas can significantly reduce their bacterial load.

## 4. Conclusions

In this work, Cu–Sn–TiO_2_ coatings were electrochemically deposited from the sulfate bath, and their microstructure and antibacterial properties were examined. The following conclusions can be drawn:

1. The results of linear polarization and chronopotentiometry experiments revealed that the introduction of 1–10 g/L of TiO_2_ particles into the sulfate electrolyte of the Cu–Sn deposition leads to a significant decrease in the cathodic current density, mainly due to reduced active electrode area involved in the electrochemical process.
2. SEM/EDX data revealed that Cu–Sn–TiO_2_ composite coatings obtained from sulfuric acid electrolyte are characterized by a more inhomogeneous structure than Cu–Sn coatings obtained under the same electrolysis conditions. It was found that TiO_2_ particles are embedded in the alloy matrix mostly in the form of agglomerates with sizes from 100 to 700 nm.
3. Electrolysis in the potentiodynamic regime allowed to effectively control the chemical composition of the metal matrix, which contained 10.8–11.5 wt.% Sn. The highest average amount of embed TiO_2_ nanoparticles (1.7 wt.%) was observed in the coating obtained from the electrolyte containing 5 g/L TiO_2_.
4. Introduction of TiO_2_ significantly improved antibacterial properties of the composites. All formed structures have pronounced bactericidal properties in relation to the strain of bacteria *E. coli*. This improvement was connected to the corrosion resistance of the formed composites. The best corrosion performance among composites was shown by the Ti-5 coating with corrosion resistance comparable to the Ti-0 coating.

## Figures and Tables

**Table 1:** Composition of the sulfate bath for electrodeposition of Cu–Sn–TiO_2_ composite coatings.

Component	Contents in Bath, g/L	Purpose of Component
CuSO_4_⋅5H_2_O	40	Source of Cu^2+^
SnSO_4_	40	Source of Sn^2+^
H_2_SO_4_	100	Base electrolyte
Thiourea	0.005	Brightening additive
TiO_2_	0–10	Second phase

**Table 2:** EDX chemical composition (scans are 50 × 50 µm^2^) of Cu–Sn and Cu–Sn–TiO_2_ coatings deposited from sulfate electrolyte with varying TiO_2_ content.

Coating	Chemical Composition, wt.%		
	Cu	Sn	TiO_2_
Ti-0	88.6 ± 0.4	11.4 ± 0.4	0
Ti-1	89.0 ± 0.5	10.8 ± 0.4	0.2 ± 0.1
Ti-5	87.8 ± 0.6	11.5 ± 0.4	1.7 ± 0.2
Ti-10	88.1 ± 0.5	11.3 ± 0.4	0.6 ± 0.1

**Table 3:** Parameters of corrosion process extracted from potentiodynamic polarization curves.

Coating	3% NaCl		Bacterial Media	
	*E*_corr_/V	*i*_corr_/10^–7^ A/cm^2^	*E*_corr_/V	*i*_corr_/10^–8^ A/cm^2^
Ti-0	0.030 ± 0.005	1.20 ± 0.11	0.064 ± 0.010	3.04 ± 0.12
Ti-1	0.019 ± 0.004	10.71± 0.09	0.099 ± 0.009	5.78 ± 0.10
Ti-5	0.043 ± 0.002	8.45 ± 2.07	0.057 ± 0.011	5.80 ± 0.24
Ti-10	0.019 ± 0.004	9.62 ± 0.13	0.045 ± 0.010	3.15 ± 0.26

**Table 4:** Fitting data and calculated polarization resistance (*R*_p_) extracted from EIS measurements.

Coating	*R*_1_, Ω∙cm^2^	*R*_2_, Ω∙cm^2^	*Y*_1_, 10^–4^ Ω^–1^ cm^–2^ s*^n^*	*n*_1_	*Y*_2_, 10^−2^ Ω^–1^ cm^–2^ s*^n^*	*n*_2_	*R*_3_, Ω∙cm^2^	*R*_p_, Ω∙cm^2^
After 15 min of corrosion test								
Ti-0	10.58 ± 4.21	1850 ± 120	2.64 ± 1.10	0.74 ± 0.01	1.34 ± 0.20	0.90 ± 0.02	5010 ± 298	6860
Ti-1	7.76 ± 5.53	1250 ± 225	4.26 ± 1.06	0.74 ± 0.02	2.12 ± 0.52	0.90 ± 0.04	2988 ± 327	4238
Ti-5	8.12 ± 3.77	1600 ± 165	2.42 ± 0.99	0.74 ± 0.01	1.16 ± 0.17	0.75 ± 0.07	4510 ± 465	6110
Ti-10	8.03 ± 2.96	1160 ± 144	4.54 ± 1.50	0.73 ± 0.03	1.22 ± 0.18	0.62 ± 0.03	2498 ± 143	3658
After 168 h of corrosion test								
Ti-0	10.21 ± 1.22	4625 ± 465	2.22 ± 0.41	0.76 ± 0.04	1.40 ± 0.28	0.70 ± 0.06	6310 ± 631	10935
Ti-1	4.18 ± 2.25	2852 ± 204	3.32 ± 1.03	0.79 ± 0.01	2.00 ± 0.92	0.69 ± 0.05	3818 ± 274	6670
Ti-5	15.98 ± 5.04	3927 ± 134	3.20 ± 0.48	0.71 ± 0.05	1.20 ± 0.09	0.60 ± 0.02	6125 ± 551	10052
Ti-10	12.94 ± 3.28	3766 ± 316	3.19 ± 1.16	0.71 ± 0.03	1.80 ± 0.66	0.64 ± 0.04	5652 ± 502	9891

## Data Availability

The raw/processed data required to reproduce the findings of this study are available from the corresponding authors upon reasonable request.

## Supplementary Materials

The following are available online at https://www.mdpi.com/article/10.3390/ma14206179/s1, Figure S1: Cross-sectional images of (a) Ti-0, (b) Ti-1, (c) Ti-5, and (d) Ti-10 composite coating. The dashed line indicates the substrate (bottom) and the coating (top) border; Figure S2: EDX elemental distribution maps of Ti-0 (a) and Ti-5 (b) coatings after 168 h of corrosion experiments in 3% NaCl solution; Table S1: Luster and microhardness of Cu–Sn and Cu–Sn–TiO_2_ coatings; Table S2: XPS surface chemical analysis of Cu–Sn and Cu–Sn–TiO_2_ coatings; Table S3: Composition of bacteria medium.

## References

[1] Bright K.R., Boone S.A., Gerba C.P. (2010). Occurrence of Bacteria and Viruses on Elementary Classroom Surfaces and the Potential Role of Classroom Hygiene in the Spread of Infectious Diseases. J. Sch. Nurs..

[2] Reynolds K.A., Watt P.M., Boone S.A., Gerba C.P. (2005). Occurrence of bacteria and biochemical markers on public surfaces. Int. J. Environ. Health Res..

[3] Boone S.A., Gerba C.P. (2007). Significance of Fomites in the Spread of Respiratory and Enteric Viral Disease. Appl. Environ. Microbiol..

[4] Mikolay A., Huggett S., Tikana L., Grass G., Braun J., Nies D.H. (2010). Survival of bacteria on metallic copper surfaces in a hospital trial. Appl. Microbiol. Biotechnol..

[5] Dalecki A.G., Crawford C.L., Wolschendorf F. (2017). Copper and Antibiotics: Discovery, Modes of Action, and Opportunities for Medicinal Applications. Advances in Microbial Physiology.

[6] Grass G., Rensing C., Solioz M. (2011). Metallic Copper as an Antimicrobial Surface. Appl. Environ. Microbiol..

[7] Walkowicz M., Osuch P., Smyrak B., Knych T., Rudnik E., Cieniek Ł., Różańska A., Chmielarczyk A., Romaniszyn D., Bulanda M. (2018). Impact of oxidation of copper and its alloys in laboratory-simulated conditions on their antimicrobial efficiency. Corros. Sci..

[8] Weaver L., Michels H.T., Keevil C.W. (2008). Survival of Clostridium difficile on copper and steel: Futuristic options for hospital hygiene. J. Hosp. Infect..

[9] Różańska A., Chmielarczyk A., Romaniszyn D., Sroka-Oleksiak A., Bulanda M., Walkowicz M., Osuch P., Knych T. (2017). Antimicrobial Properties of Selected Copper Alloys on Staphylococcus aureus and Escherichia coli in Different Simulations of Environmental Conditions: With vs. without Organic Contamination. Int. J. Environ. Res. Public Health.

[10] Montero D.A., Arellano C., Pardo M., Vera R., Gálvez R., Cifuentes M., Berasain M.A., Gómez M., Ramírez C., Vidal R.M. (2019). Antimicrobial properties of a novel copper-based composite coating with potential for use in healthcare facilities. Antimicrob. Resist. Infect. Control.

[11] Chang T., Butina K., Herting G., Rajarao G.K., Richter-Dahlfors A., Blomberg E., Odnevall Wallinder I., Leygraf C. (2021). The interplay between atmospheric corrosion and antimicrobial efficiency of Cu and Cu5Zn5Al1Sn during simulated high-touch conditions. Corros. Sci..

[12] Van Doremalen N., Bushmaker T., Morris D.H., Holbrook M.G., Gamble A., Williamson B.N., Tamin A., Harcourt J.L., Thornburg N.J., Gerber S.I. (2020). Aerosol and Surface Stability of SARS-CoV-2 as Compared with SARS-CoV-1. N. Engl. J. Med..

[13] Huang H., Fan C., Li M., Nie H.-L., Wang F.-B., Wang H., Wang R., Xia J., Zheng X., Zuo X. (2020). COVID-19: A Call for Physical Scientists and Engineers. ACS Nano.

[14] Hutasoit N., Kennedy B., Hamilton S., Luttick A., Rahman Rashid R.A., Palanisamy S. (2020). Sars-CoV-2 (COVID-19) inactivation capability of copper-coated touch surface fabricated by cold-spray technology. Manuf. Lett..

[15] Chang T., Sepati M., Herting G., Leygraf C., Rajarao G.K., Butina K., Richter-Dahlfors A., Blomberg E., Odnevall Wallinder I. (2021). A novel methodology to study antimicrobial properties of high-touch surfaces used for indoor hygiene applications—A study on Cu metal. PLoS ONE.

[16] Poggio C., Colombo M., Arciola C.R., Greggi T., Scribante A., Dagna A. (2020). Copper-Alloy Surfaces and Cleaning Regimens against the Spread of SARS-CoV-2 in Dentistry and Orthopedics. From Fomites to Anti-Infective Nanocoatings. Materials.

[17] Gamburg Y.D., Zangari G. (2011). Electrodeposition of Alloys. Theory and Practice of Metal Electrodeposition.

[18] Jung M., Lee G., Choi J. (2017). Electrochemical plating of Cu-Sn alloy in non-cyanide solution to substitute for Ni undercoating layer. Electrochim. Acta.

[19] Kasach A.A., Kharitonov D.S., Romanovskii V.I., Kuz’menok N.M., Zharskii I.M., Kurilo I.I. (2019). Electrodeposition of Cu-Sn Alloy from Oxalic Acid Electrolyte in the Presence of Amine-containing Surfactants. Russ. J. Appl. Chem..

[20] Kasach A.A., Kharitonov D.S., Makarova I.V., Wrzesińska A., Zharskii I.M., Kurilo I.I. (2020). Effect of thiourea on electrocrystallization of Cu–Sn alloys from sulphate electrolytes. Surf. Coat. Technol..

[21] Juškenas R., Mockus Z., Kanapeckaite S., Stalnionis G., Survila A. (2006). XRD studies of the phase composition of the electrodeposited copper-rich Cu-Sn alloys. Electrochim. Acta.

[22] Kasach A.A., Kharitonov D.S., Radchenko S.L., Zharskii I.M., Kurilo I.I. (2020). Effect of Parameters of Pulse Electrolysis on Electrodeposition of Copper–Tin Alloy from Sulfate Electrolyte. Russ. J. Electrochem..

[23] Low C.T.J., Wills R.G.A., Walsh F.C. (2006). Electrodeposition of composite coatings containing nanoparticles in a metal deposit. Surf. Coat. Technol..

[24] Li B., Zhang W. (2020). Facile synthesis and electrochemical properties of a novel Ni-B/TiC composite coating via ultrasonic-assisted electrodeposition. Ultrason. Sonochem..

[25] Lixia Y., Zhenghui L., Ke W., Xiupeng L., Guixiang W. (2017). Effect of TiO_2_ Sol on the Microstructure and Tribological Properties of Cu-Sn Coating. Rare Met. Mater. Eng..

[26] Walsh F.C., Wang S., Zhou N. (2020). The electrodeposition of composite coatings: Diversity, applications and challenges. Curr. Opin. Electrochem..

[27] Walsh F.C., de Leon C.P. (2014). A review of the electrodeposition of metal matrix composite coatings by inclusion of particles in a metal layer: An established and diversifying technology. Trans. IMF.

[28] Cui G., Bi Q., Niu M., Yang J., Liu W. (2013). The tribological properties of bronze-SiC-graphite composites under sea water condition. Tribol. Int..

[29] Wang X.H., Ying L.X., Zhang C.J., Lv X.P. (2017). Electrodeposition of Cu-Sn-Graphite-Al2O3 Composite Coatings and their Tribological Properties. Proceedings of the Mechanical Engineering, Materials Science and Civil Engineering IV.

[30] Ying L., Fu Z., Wu K., Wu C., Zhu T., Xie Y., Wang G. (2019). Effect of TiO_2_ Sol and PTFE Emulsion on Properties of Cu–Sn Antiwear and Friction Reduction Coatings. Coatings.

[31] Kasach A.A., Kharitonov D.S., Wrzesińska A., Bobowska I., Predko A.A., Romanovskii V.I., Zharskii I.M., Kurilo I.I. (2020). The Effect of Ultrasound Treatment on Physicochemical and Tribological Properties of Electrolytic Cu–Sn–TiO_2_ Coatings. Prot. Met. Phys. Chem. Surfaces.

[32] Gao W., Cao D., Jin Y., Zhou X., Cheng G., Wang Y. (2018). Microstructure and properties of Cu-Sn-Zn-TiO_2_ nano-composite coatings on mild steel. Surf. Coat. Technol..

[33] Danilov F.I., Tsurkan A.V., Vasil’eva E.A., Korniy S.A., Cheipesh T.A., Protsenko V.S. (2017). Electrochemical synthesis and properties of iron–titanium dioxide composite coatings. Russ. J. Appl. Chem..

[34] Danilov F.I., Kityk A.A., Shaiderov D.A., Bogdanov D.A., Korniy S.A., Protsenko V.S. (2019). Electrodeposition of Ni–TiO_2_ Composite Coatings Using Electrolyte Based on a Deep Eutectic Solvent. Surf. Eng. Appl. Electrochem..

[35] Protsenko V.S., Bogdanov D.A., Korniy S.A., Kityk A.A., Baskevich A.S., Danilov F.I. (2019). Application of a deep eutectic solvent to prepare nanocrystalline Ni and Ni/TiO_2_ coatings as electrocatalysts for the hydrogen evolution reaction. Int. J. Hydrogen Energy.

[36] Wysocka I., Kowalska E., Ryl J., Nowaczyk G., Zielińska-Jurek A. (2019). Morphology, Photocatalytic and Antimicrobial Properties of TiO_2_ Modified with Mono- and Bimetallic Copper, Platinum and Silver Nanoparticles. Nanomaterials.

[37] Pyanko A.V., Makarova I.V., Kharitonov D.S., Makeeva I.S., Alisienok O.A., Chernik A.A. (2019). Tin–Nickel–Titania Composite Coatings. Inorg. Mater..

[38] Chang T., Maltseva A., Volovitch P., Odnevall Wallinder I., Leygraf C. (2020). A mechanistic study of stratified patina evolution on Sn-bronze in chloride-rich atmospheres. Corros. Sci..

[39] Kharitonov D.S., Kasach A.A., Sergievich D.S., Wrzesińska A., Bobowska I., Darowicki K., Zielinski A., Ryl J., Kurilo I.I. (2021). Ultrasonic-assisted electrodeposition of Cu-Sn-TiO_2_ nanocomposite coatings with enhanced antibacterial activity. Ultrason. Sonochem..

[40] Guglielmi N. (1972). Kinetics of the Deposition of Inert Particles from Electrolytic Baths. J. Electrochem. Soc..

[41] Bengoa L.N., Pary P., Conconi M.S., Egli W.A. (2017). Electrodeposition of Cu-Sn alloys from a methanesulfonic acid electrolyte containing benzyl alcohol. Electrochim. Acta.

[42] Biesinger M.C. (2017). Advanced analysis of copper X-ray photoelectron spectra. Surf. Interface Anal..

[43] Kubacka A., Diez M.S., Rojo D., Bargiela R., Ciordia S., Zapico I., Albar J.P., Barbas C., Martins Dos Santos V.A.P., Fernández-García M. (2014). Understanding the antimicrobial mechanism of TiO_2_-based nanocomposite films in a pathogenic bacterium. Sci. Rep..

[44] Hutchison M.J., Scully J.R. (2018). Patina enrichment with SnO_2_ and its effect on soluble Cu cation release and passivity of high-purity Cu-Sn bronze in artificial perspiration. Electrochim. Acta.

[45] Hutchison M.J., Zhou P., Ogle K., Scully J.R. (2017). Enhanced Electrochemical Cu Release from Commercial Cu-Sn Alloys: Fate of the Alloying Elements in Artificial Perspiration. Electrochim. Acta.

[46] Da Silva F.S., Cinca N., Dosta S., Cano I.G., Guilemany J.M., Caires C.S.A., Lima A.R., Silva C.M., Oliveira S.L., Caires A.R.L. (2019). Corrosion resistance and antibacterial properties of copper coating deposited by cold gas spray. Surf. Coat. Technol..

[47] Tudela I., Zhang Y., Pal M., Kerr I., Cobley A.J. (2014). Ultrasound-assisted electrodeposition of composite coatings with particles. Surf. Coat. Technol..

[48] Bahadormanesh B., Dolati A., Ahmadi M.R. (2011). Electrodeposition and characterization of Ni–Co/SiC nanocomposite coatings. J. Alloy. Compd..

