# Supplementary material for: Effect of TiO2 Concentration on Microstructure and Properties of Composite Cu–Sn–TiO2 Coatings Obtained by Electrodeposition
Source: Materials (Basel). 2021 Oct 18;14(20):6179. doi: 10.3390/ma14206179 (PMC8540675; doi:10.3390/ma14206179)
Supplement: Supplementary file 1 [file materials-14-06179-s001.zip › materials-1401458-supplementary.pdf]

materials

Effect of TiO_2 Concentration on Microstructure and Properties of Composite Cu–Sn–TiO_2 Coatings Obtained by Electrodeposition

Aliaksandr A. Kasach [1,*], Dzmitry S. Kharytonau [2,*], Andrei V. Paspelau [3], Jacek Ryl [4], Denis S. Sergievich [5], Ivan M. Zharskii [1] and Irina I. Kurilo [6]

1 Department of Chemistry, Electrochemical Production Technology and Materials for Electronic Equipment, Chemical Technology and Engineering Faculty, Belarusian State Technological University, Sverdlova 13a, 220006 Minsk, Belarus; inorgchem@belstu.by
2 Jerzy Haber Institute of Catalysis and Surface Chemistry, Polish Academy of Sciences, Niezapominajek 8, 30-239 Krakow, Poland
3 Physical and Chemical Investigations Methods Center, Belarusian State Technological University, Sverdlova 13a, 220006 Minsk, Belarus; pospelov.ip@gmail.com
4 Institute of Nanotechnology and Materials Engineering, Faculty of Applied Physics and Mathematics, Gdansk University of Technology, 80-233 Gdansk, Poland; jacek.ryl@pg.edu.pl
5 Department of Biotechnology, Organic Substances Technology Faculty, Belarusian State Technological University, Sverdlova 13a, 220006 Minsk, Belarus; sergievich@belstu.by
6 Department of Physical, Colloid and Analytical Chemistry, Organic Substances Technology Faculty, Belarusian State Technological University, Sverdlova 13a, 220006 Minsk, Belarus; kurilo@belstu.by
* Correspondence: kasach2018@bk.ru (A.A.K.); dmitry.kharitonov@ikifp.edu.pl (D.S.K.)

Citation: Kasach, A.A.; Kharytonau, D.S.; Paspelau, A.V.; Ryl, J.; Sergievich, D.S.; Zharskii, I.M.; Kurilo, I.I. Effect of TiO_2 Concentration on Microstructure and Properties of Composite Cu–Sn–TiO_2 Coatings Obtained by Electrodeposition. *Materials* **2021**, *14*, 6179. https://doi.org/10.3390/ma14206179

Academic Editor: Zbigniew Brytan

Received: 14 September 2021
Accepted: 14 October 2021
Published: 18 October 2021

Publisher's Note: MDPI stays neutral with regard to jurisdictional claims in published maps and institutional affiliations.

Copyright: © 2021 by the authors. Licensee MDPI, Basel, Switzerland. This article is an open access article distributed under the terms and conditions of the Creative Commons Attribution (CC BY) license (http://creativecommons.org/licenses/by/4.0/).

Scheme 1. Microhardness and visual appearance.

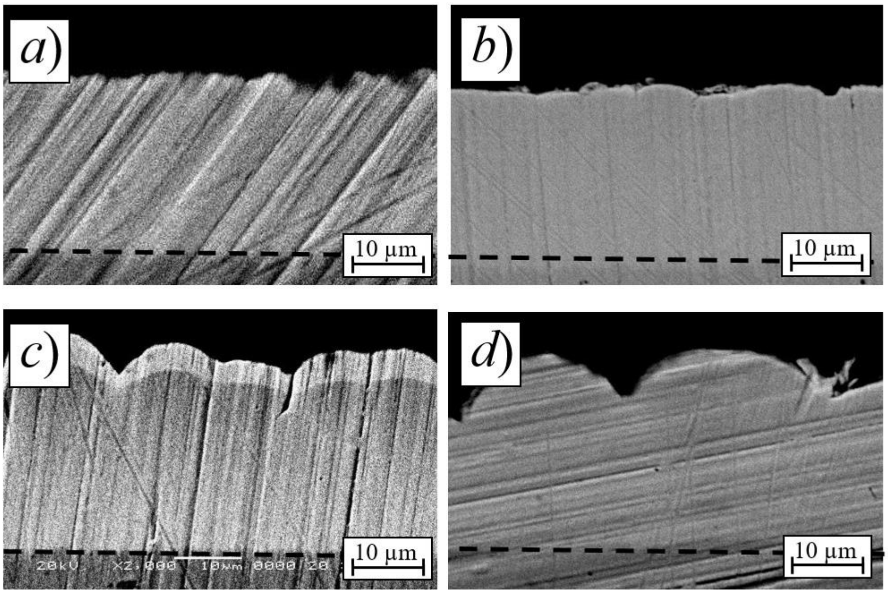

Figure S1. Cross-sectional images of (**a**) Ti-0, (**b**) Ti-1, (**c**) Ti-5, and (**d**) Ti-10 composite coating. The dashed line indicates the substrate (bottom) and the coating (top) border.

The microhardness of the obtained coatings was measured using an AFFRI–MVDM8 hardness tester. Before the deposition of the studied alloys, 1 μm of copper from a pyrophosphate electrolyte (pH 9) of the following composition, mol/L: 0.44 $Cu_2P_2O_7$, 1.00 $K_4P_2O_7$, 0.04 $C_3H_8O_3$, was deposited on the surface. The thickness of the examined coatings was 15 μm.

The luster of the obtained coatings was examined using a photoelectric gloss meter BF 2 M–45/0/45. The luster values are given relative to a silver mirror.

Table S1 shows the results of measuring the microhardness and luster of the obtained coatings. The microhardness of the Ti–0 coatings was 269.6 HV. Modification of coatings with TiO_2 particles leads to an increase in their microhardness. The highest microhardness values were observed for Ti–5 coatings, which have the highest TiO_2 content. TiO_2 particles incorporated into the alloy matrix prevent the movement of dislocations in the coating structure, which leads to an increase in their microhardness.

Semi-lustrous coatings with a luster of 43.4% are formed from the sulfate electrolyte that does not contain TiO_2 particles. The introduction of a dispersed phase into the electrolyte composition leads to a significant decrease in the luster of the formed coatings, which decreased from 30.1 to 18.7% for Ti–1 and Ti–10 coatings, respectively. The decrease in the reflectivity of the coatings with an increase in the TiO_2 content is due to the more inhomogeneous surface morphology of the formed coatings.

Table S1. Luster and microhardness of Cu–Sn and Cu–Sn–TiO_2 coatings.

Coating	Luster, %	Microhardness, HV
Ti-0	43.7 ± 3.3	269.6 ± 15.1
Ti-1	30.1 ± 4.1	302.6 ± 19.6
Ti-5	26.5 ± 4.2	320.4 ± 15.6
Ti-10	18.7 ± 2.1	316.4 ± 22.6

S2. XPS analysis

Table S2. The XPS surface chemical analysis of Cu–Sn and Cu–Sn–TiO_2 coatings.

	Cu2p3/2		Sn3d5/2	Ti2p3/2
	Cu^+	Cu^{2+}	Sn^{4+}	Ti^{4+}
BE / eV	932.7	934.1	486.6	458.4
Cu–Sn, at%	56.4	41.2	2.5	–
Cu–Sn–TiO_2, at%	53.4	32.8	10.7	3.1

S3. Corrosion analysis in NaCl solution.

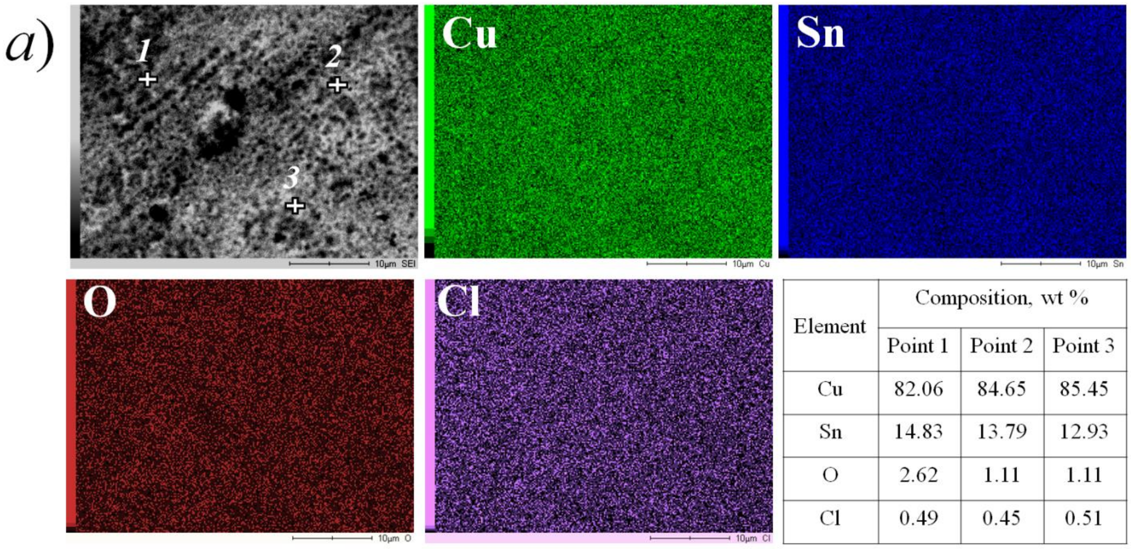

Element	Composition, wt %		
	Point 1	Point 2	Point 3
Cu	82.06	84.65	85.45
Sn	14.83	13.79	12.93
O	2.62	1.11	1.11
Cl	0.49	0.45	0.51

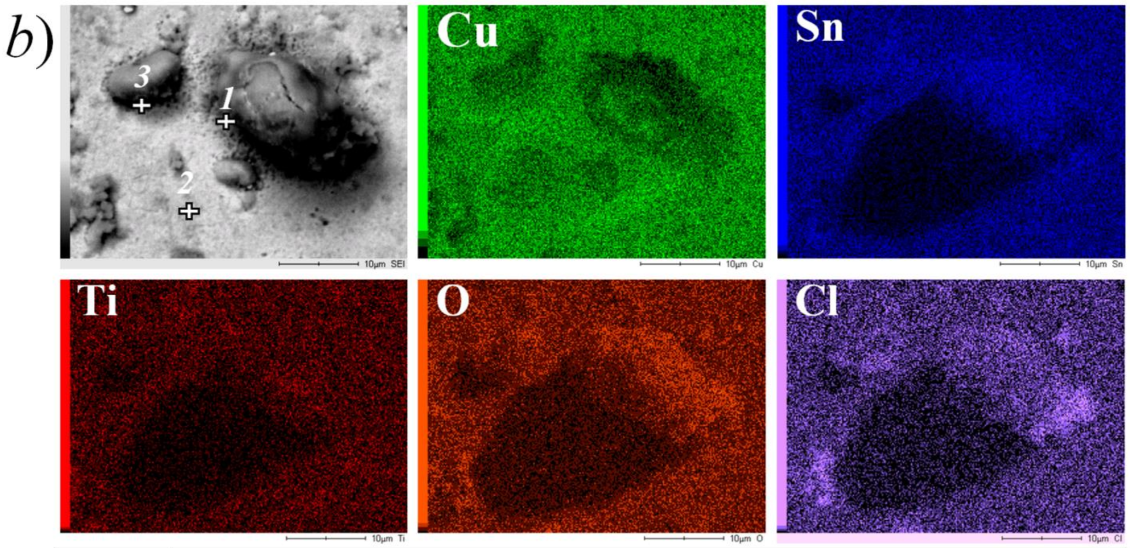

Point	Composition, wt %				
	Element				
	Cu	Sn	Ti	O	Cl
1	71.01	20.79	0.54	7.02	0.63
2	79.28	15.66	0.20	4.27	0.59
3	74.50	19.23	0.44	5.24	0.6

Figure S2. EDX elemental distribution maps of Ti-0 (**a**) and Ti-5 (**b**) coatings after 168 h of corrosion experiments in 3% NaCl solution.

S4. Corrosion analysis in bacterial media.

The composition of the examined medium is presented in Table S3.

Table S3. Composition of bacteria medium.

Component	Composition, g/L
Fishmeal pancreatic hydrolysate	8
Enzymatic peptone	8
NaCl	4
pH	7.0–7.4
